# Virtual non-contrast images from dual-layer spectral CT: comparison with true non-contrast across abdominal structures

**DOI:** 10.1007/s00330-026-12642-0

**Published:** 2026-06-03

**Authors:** Domenico De Santis, Antonella Del Gaudio, Benedetta Masci, Bianca Catalano, Marta Zerunian, Michela Polici, Francesco Pucciarelli, Giuseppe Tremamunno, Tiziano Polidori, Ettore Squillaci, Cesare Maino, Davide Ippolito, Marco Francone, Damiano Caruso

**Affiliations:** 1 Department of Medical-Surgical Sciences and Translational Medicine, School of Medicine and Psychology, Sant’Andrea University Hospital, Sapienza University of Rome, Rome, Italy; 2 PhD School in Translational Medicine and Oncology, Department of Medical and Surgical Sciences and Translational Medicine, Faculty of Medicine and Psychology, Sapienza University of Rome, Rome, Italy; 3 Unità Operativa Complessa di Diagnostica per Immagini e Radiologia Interventistica, Ospedale Isola Tiberina-Gemelli Isola, Rome, Italy; 4 Department of Diagnostic Radiology, Fondazione IRCCS San Gerardo dei Tintori, Monza, Italy

**Keywords:** Abdomen, Calcifications, Spectral CT, Computed tomography, Virtual non-contrast imaging

## Abstract

**Objective:**

To compare virtual non-contrast (VNC) images derived from arterial (VNC_A) and portal venous phase (VNC_P) of dual-layer spectral CT (DLCT) with true non-contrast (TNC) images in abdominal imaging, assessing attenuation accuracy, image quality, lesion and calcification measurements, and the potential of VNC to replace TNC in routine protocols.

**Materials and methods:**

One hundred consecutive patients undergoing triphasic abdominal CT were included. TNC, arterial, and portal venous phases were acquired on a DLCT scanner. VNC_A and VNC_P images were generated using a two-material decomposition algorithm. Attenuation values, image noise, signal-to-noise ratio (SNR), contrast-to-noise ratio (CNR), and lesion and calcification metrics were measured. Subjective image quality and diagnostic acceptability were graded by three radiologists. Agreement with TNC was evaluated using ICC, Bland–Altman analysis, ANOVA, and non-parametric tests. Radiation dose reduction achievable by omitting the TNC phase was also assessed.

**Results:**

Although VNC_A showed better agreement with TNC than VNC_P, both consistently underestimated parenchymal and lesion attenuation; lesion size was preserved (12.2 ± 7.9 mm; *p* = 0.996). Calcification density was significantly reduced in both VNC phases, and calcification diameter was slightly underestimated in VNC_A (*p* < 0.001). VNC images demonstrated higher or similar SNR and CNR but inferior subjective image quality (*p* < 0.001), due to blotchier appearance. Only 12% of VNC_P datasets were judged fully interchangeable with TNC. Replacing TNC with VNC would yield a 33% radiation dose reduction in this specific protocol.

**Conclusions:**

VNC cannot replace TNC based on the current evidence; further refinements are required for broader clinical adoption.

**Key Points:**

***Question*** *True non-contrast images (TNC) are essential in abdominal CT, yet it remains unclear whether spectral CT-derived virtual non-contrast images (VNC) can reliably replace TNC imaging.*

***Findings*** *VNC images approximate TNC images but introduce consistent attenuation biases, reduced subjective quality, and altered calcification metrics, preventing full replacement of dedicated non-contrast acquisitions.*

***Clinical relevance*** *Replacing TNC with VNC images reduces radiation exposure, yet current VNC performance shows measurable differences from TNC, indicating that algorithmic refinement is required before routine diagnostic use in abdominal CT.*

**Graphical Abstract:**

**Figure:**
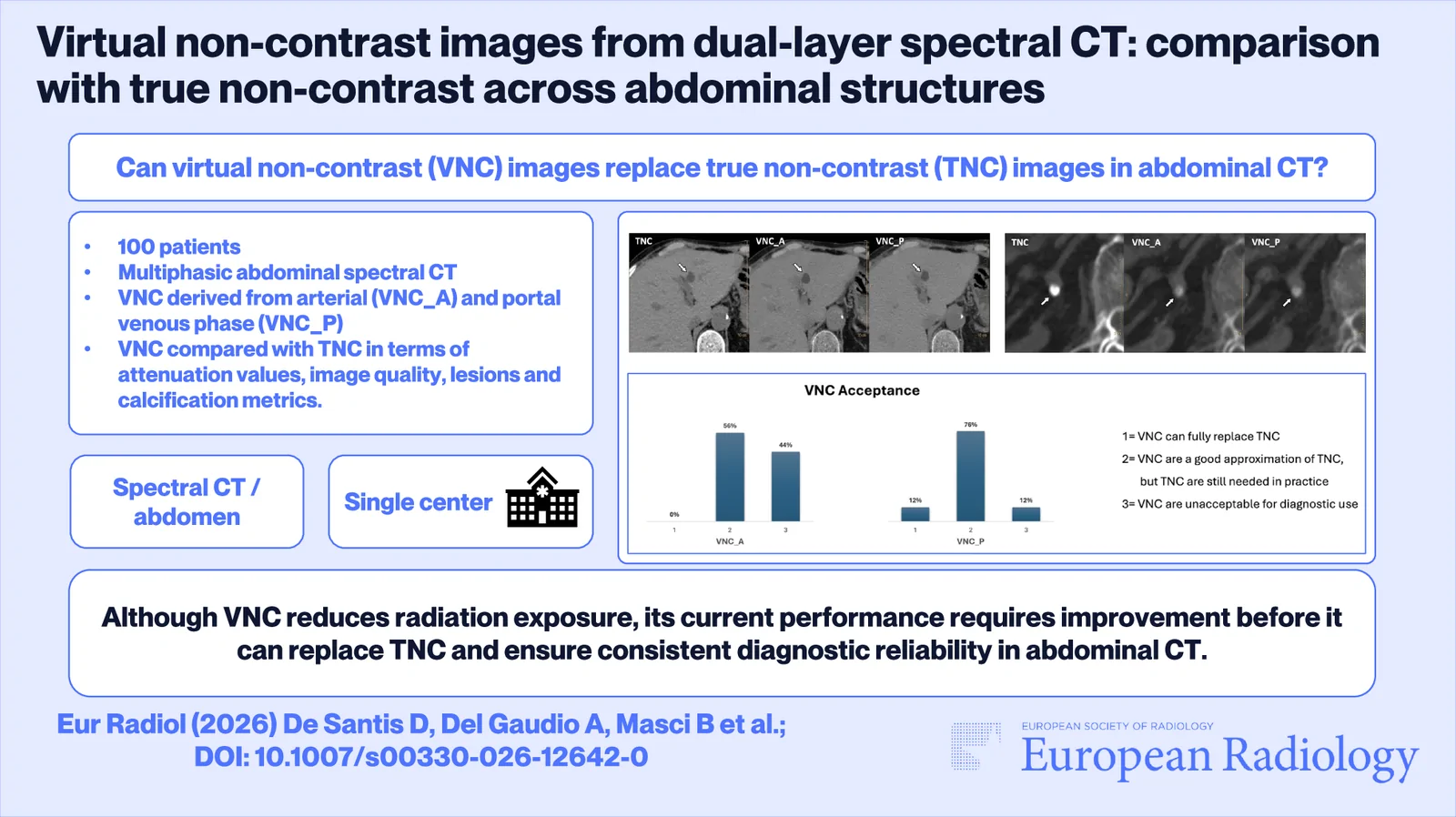

## Introduction

True non-contrast (TNC) phase is routinely acquired in multiphasic abdominal CT studies due to its role in characterizing parenchymal lesions, detecting hemorrhage and calcification; nevertheless, its acquisition plays a substantial role in the total scan time and radiation exposure. In recent years, the advent of spectral CT technology has allowed for the reconstruction of virtual non-contrast (VNC) images from contrast-enhanced datasets. VNC images are generated by subtracting the iodine component from contrast-enhanced images, aiming at simulating the TNC images while potentially reducing the need for a dedicated non-enhanced acquisition [1, 2]. While previous studies have shown promising results for VNC imaging in different clinical settings, evidence regarding the accuracy of VNC images in adequately simulating TNC images for lesion detection and quantitative analysis is still mixed and technology-dependent [3–6]. In particular, most data were obtained with dual-source CT, rapid kVp-switching CT systems, and more recently, with photon-counting CT technology, whereas fewer studies have focused on dual-layer spectral detector CT (DLCT) scanners [7–10]. This technology is based on a single conventional x-ray tube coupled with a layered detector that separates high-energy and low-energy photons, without temporal or spatial misregistration [4]. DLCT theoretically allows robust spectral separation across all anatomical regions and acquisition modes, making it potentially well-suited for abdominal imaging, a field where accurate lesion measurement and consistent attenuation assessment are critical, especially in oncology and follow-up imaging. For the VNC dataset to replace TNC in abdominal CT protocols, it must ensure reliable lesion characterization, accurate calcification assessment, and consistent measurements during longitudinal follow-up, particularly in oncologic settings. In addition, subjective image quality is an important determinant of diagnostic confidence and is crucial in determining whether VNC can be implemented in routine clinical practice. Although prior studies on DLCT have demonstrated good agreement between portal phase–derived VNC and TNC images in normal abdominal parenchyma, the performance of VNC across different acquisition phases, particularly with respect to lesion size stability, calcification quantification, and subjective image quality, has not been comprehensively investigated. Therefore, the aim of this study was to compare arterial- and portal venous phase–derived VNC images from DLCT with standard TNC images in terms of image quality and abdominal lesions and calcifications measurements, in order to evaluate the potential of VNC images to replace TNC images in routine abdominal CT protocols.

## Materials and methods

### Patient population

This prospective single-center study was conducted from December 2024 to February 2025, in accordance with the local Institutional Review Board. Consecutive inpatients performing routine triphasic abdominal CT were included. Informed consent was signed by all participants before collecting personal data and undergoing CT examinations.

Patients with clinically indicated abdominal CT were included in the study. Exclusion criteria included age under 18, pregnancy, impaired kidney function (estimated glomerular filtration rate < 30 mL/min/1.73 m^2^), contraindications to iodinated contrast medium administration, severe steatosis (liver attenuation on unenhanced images of < 40 HU), cirrhosis, and liver affected by diffuse lesions preventing the placement of regions of interest (ROIs). Patients’ weight and height were recorded, and body mass index (BMI) was calculated.

### CT image acquisition

All CT examinations were performed on a single DLCT scanner (Philips 7500, Philips Healthcare). Patients were examined in a supine position, with arms raised above the head. The acquisition protocol consisted of a triphasic examination including a TNC phase, contrast-enhanced arterial phase, and portal venous phase. All acquisitions were performed with the following parameters: detector collimation, 128 × 0.625; pitch, 0.800; rotation time, 0.33 s; tube voltage, 120 kVp. Automated tube current modulation (DoseRight, Philips Healthcare) was applied with the following parameters: dose right index (DRI), 21; liver area DRI, +5. After the acquisition of the TNC phase, scan delay was determined with a dedicated bolus-tracking technique by placing an ROI with a threshold of 100 HU within the abdominal aorta below the diaphragmatic dome; the arterial and portal images were acquired with a 15-s and a 60-s delay, respectively.

Parenchymal and vascular enhancement was obtained with administration of non-ionic iodinated contrast medium (Iomeprol, Iomeron 400; Bracco Imaging) at a flow rate of 3.5 mL/s, followed by an injection of 30 mL saline flush through a superficial vein access in the antecubital fossa at the same flow rate. The amount of contrast medium was based on lean-body weight evaluation using James formula [11]. All scans covered a field of view comprising the diaphragm to the symphysis pubis.

### CT image reconstruction

All image data were reconstructed with a model-based iterative reconstruction algorithm (IMR, Philips Healthcare) at strength level 1, routine kernel, section thickness of 2 mm and increment of 1 mm. Image data were then transferred to a dedicated multi-modality post-processing system (Philips Advanced Visualization Workspace, Philips Healthcare) and loaded into the dedicated dual-energy elaborating system.

DLCT performs projection-domain material decomposition using energy-dependent attenuation differences modeled through photoelectric and Compton basis functions, enabling two-material basis decomposition. In clinical abdominal applications, an iodine–water basis pair is typically employed [12]. Accordingly, VNC images derived from arterial phase (VNC_A) and portal venous phase (VNC_P) acquisitions were generated by subtracting the iodine contribution from the contrast-enhanced datasets; all spectral reconstructions were performed using the vendor’s default settings, without parameter modifications. For the purpose of this study, VNC_A, VNC_P, and TNC datasets were further evaluated.

### Objective image analysis

All images were reviewed by a single radiologist with 5 years of experience in abdominal imaging on a dedicated workstation (Philips Advanced Visualization Workspace, Philips Healthcare). Circular ROIs sized 0.4 cm^2^ to 1 cm^2^ were placed in (1) the liver parenchyma; (2) portal vein; (3) upper abdominal aorta, avoiding the vessel wall; (4) renal cortex at the mid‑renal level; (5) central splenic parenchyma away from the hilum and capsule; (6) visually normal pancreatic parenchyma of the head/body while carefully excluding the main pancreatic duct, intrapancreatic vessels, focal lesions, and peripancreatic fat; (7) paravertebral back muscles at L2 level; (8) and within the retroperitoneal fat at the same level. Average attenuation values expressed in Hounsfield Units (HU) were recorded. To determine liver attenuation values, three ROIs were placed in the II, IV, and VI segments, avoiding focal lesions biliary tree or large intrahepatic vessels; attenuation values were eventually averaged. Copy-paste function was applied to ensure consistency in ROI placement among the three datasets.

Additionally, the attenuation values of each hypoattenuating liver, spleen, or kidney lesion were recorded. Image noise was assessed as the standard deviation (SD) of the ROIs placed in the retroperitoneal fat.

Signal-to-noise ratio (SNR) was calculated as follows:

$${SNR}=\frac{{{HU}}_{{parenchyma}}}{{{SD}}_{{fat}}}$$

Contrast-to-noise ratio (CNR) was calculated as follows:

$${CNR}=\frac{({{HU}}_{{parenchyma}}-\,{{HU}}_{{muscle}})}{{{SD}}_{{fat}}}$$

### Subjective image analysis

Subjective image quality was independently assessed in the three datasets by two radiologists with 5 and 6 years of experience in abdominal-CT imaging, blinded to the reconstruction algorithm; discrepancies were resolved in consensus by a third reader with 10 years of experience in abdominal imaging. Consensus scores were used for final analysis. The analysis was performed in random order and with a time interval of 10 days between the three datasets, to avoid recall bias.

Image noise and the overall image quality were assessed in TNC, VNC_A, and VNC_P reconstructions; VNC_A and VNC_P were also subjectively evaluated for calcium subtraction and lesion conspicuity. Subjective image quality scores were graded with a 4-point Likert scale, where 1 was the best score and 4 was the worst score for all determinants (Supplemental Table 1). Additionally, readers graded VNC_A and VNC_P acceptance with a 3-point Likert scale: 1, VNC can fully replace TNC; 2, VNC is a good approximation of TNC, but TNC is still needed in clinical practice; 3, VNC is unacceptable for diagnostic use [13].

### Lesions and calcifications analysis

The same three readers in consensus marked all visible ≥ 3 mm hypoattenuating liver, spleen, and kidney lesions, as well as vascular calcifications and kidney stones in the three datasets. Attenuation values were recorded by placing circular ROIs, and the copy-paste function was also applied to ensure consistency in ROI placement. Lesions and calcifications size were measured; each measurement was performed three times and then averaged.

### Radiation dose

The dose-length product (DLP) of each phase was recorded for each examination. Effective radiation dose (ED) was calculated by multiplying the DLP by an abdomen-specific conversion coefficient k of 0.015 mSv·mGy^−1^·cm^−1^ [14]. Although recent Monte Carlo simulation studies suggest that gender-specific k-factors for abdominal dual-energy CT may differ slightly from the conventional value [15], the standard coefficient was used to ensure comparability with prior literature.

### Statistical analysis

Statistical analysis was performed by using commercially available software (IBM Corp., Released 2019. IBM SPSS Statistics for Windows, Version 26.0., IBM Corp). Normal distribution of continuous data was assessed with the Kolmogorov–Smirnov test. Continuous variables are reported as mean ± standard deviation, while ordinal data are reported as median and interquartile range (IQR).

Agreement between parenchymal and lesion attenuation values (HU) measured on TNC, VNC_A, and VNC_P was assessed with quantitative tests and graphical methods. The Intraclass Correlation Coefficient (ICC) was calculated using a two-way random-effects model for absolute agreement [ICC [2,1]], as this model is recommended when assessing agreement between different measurement methods and accounts for both systematic bias and random error. ICC values were interpreted according to standard thresholds: poor (< 0.5), moderate (0.5–0.75), good (0.75–0.90), and excellent (> 0.90) agreement [16]. Additionally, Bland–Altman plots were constructed for each organ to visualize bias and 95% limits of agreement (LOA) between TNC and both VNC_A and VNC_P.

A one-way repeated measures ANOVA with Bonferroni post hoc correction was conducted to determine whether there was a statistically significant difference in attenuation values and objective image quality among TNC, VNC_A, and VNC_P datasets.

Friedman’s test and the Wilcoxon signed-rank tests were used to compare differences on subjective image quality.

A 2-tailed *p*-level of < 0.05 indicated a statistically significant difference in the tests performed.

## Results

### Patient population

From an initial population of 109 patients, a total of 9 individuals were excluded due to severe liver steatosis (*n* = 7), cirrhosis (*n* = 1), and liver affected by diffuse lesions (*n* = 1). Hence, the final population consisted of 100 patients (males: 68; age: 67.5 ± 13.1 years; range: 31–95 years). BMI (24.9 ± 4.3; range: 15.8–36.8). Mean injected contrast medium volume was 93.0 ± 18.3 mL (range: 45–135 mL).

### Attenuation values

**Fig. 1:**
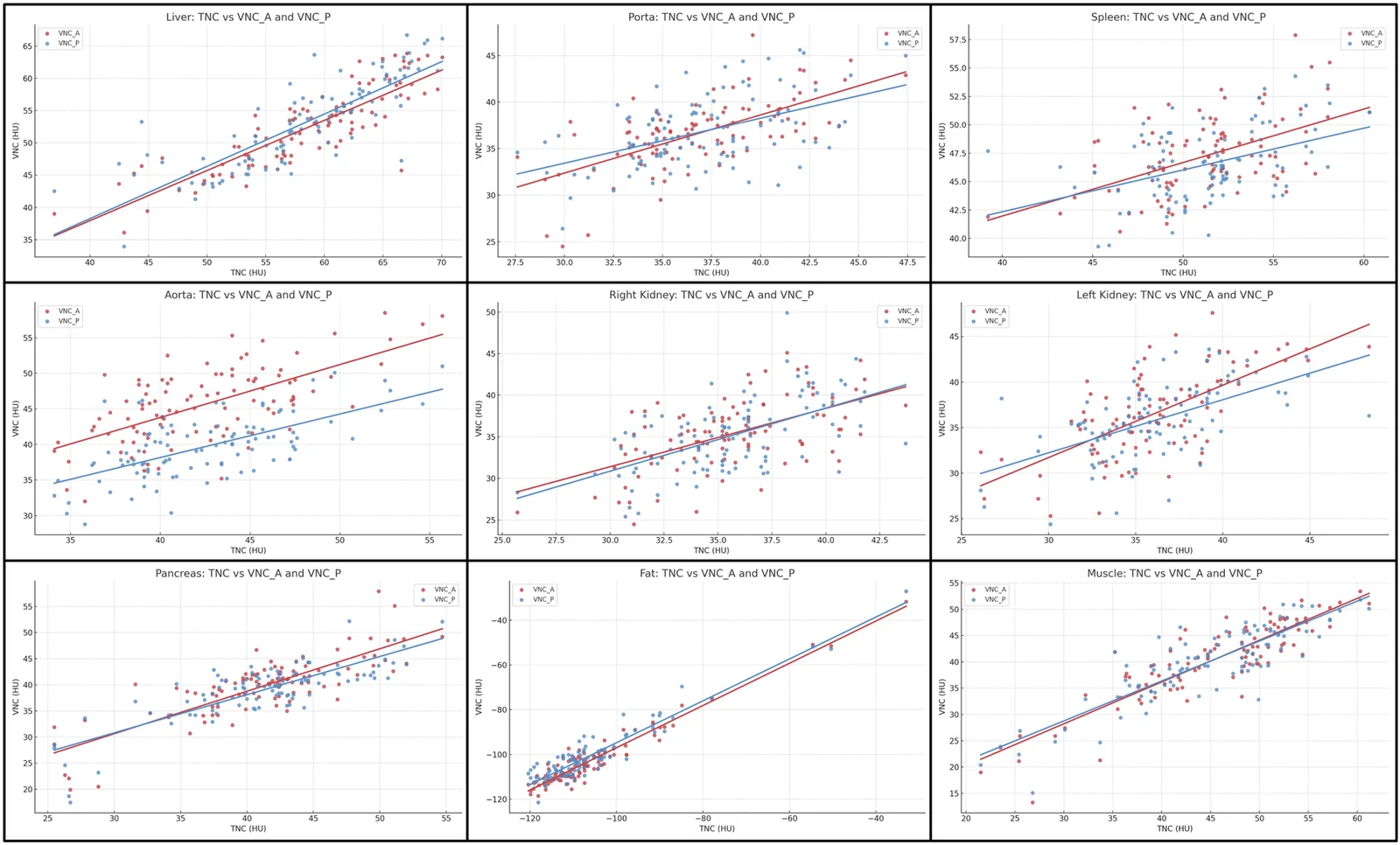
Scatterplots comparing attenuation values (HU) measured on TNC images versus VNC_A (in red) and VNC_P (in blue) across different abdominal organs. Each point represents a patient-level measurement. Regression lines are shown for both VNC phases

Table 1 shows the ICC values for each abdominal structure for both VNC_A and VNC_P datasets; corresponding scatterplots are depicted in Fig. 1. VNC_A demonstrated overall superior agreement with TNC compared to VNC_P. Specifically, VNC_A showed higher ICC values in liver (0.855 vs 0.829), portal vein (0.653 vs 0.485), spleen (0.509 vs 0.427), pancreas (0.816 vs 0.806), left kidney (0.650 vs 0.560), fat (0.964 vs 0.946), and muscle (0.873 vs 0.859).

**Table 1:** ICC values for each abdominal structure for both VNC_A and VNC_P datasets

	TNC vs VNC_A		TNC vs VNC_P	
	ICC	95% CI	ICC	95% CI
Liver	0.855	0.791–0.912	0.829	0.755–0.882
Porta	0.653	0.523–0.753	0.485	0.358–0.593
Aorta	0.636	0.502–0.740	0.637	0.498–0.735
Spleen	0.509	0.382–0.604	0.427	0.301–0.526
Right kidney	0.549	0.407–0.661	0.564	0.413–0.685
Left kidney	0.650	0.519–0.751	0.560	0.408–0.682
Pancreas	0.816	0.737–0.873	0.806	0.724–0.866
Fat	0.964	0.947–0.976	0.946	0.920–0.964
Muscle	0.873	0.816–0.913	0.859	0.797–0.903

*TNC* true non-contrast, *VNC_A* virtual non-contrast derived from arterial phase, *VNC_P* virtual non-contrast derived from portal venous phase, *ICC* intraclass correlation coefficient, *95% CI* 95% confidence interval

**Fig. 2:**
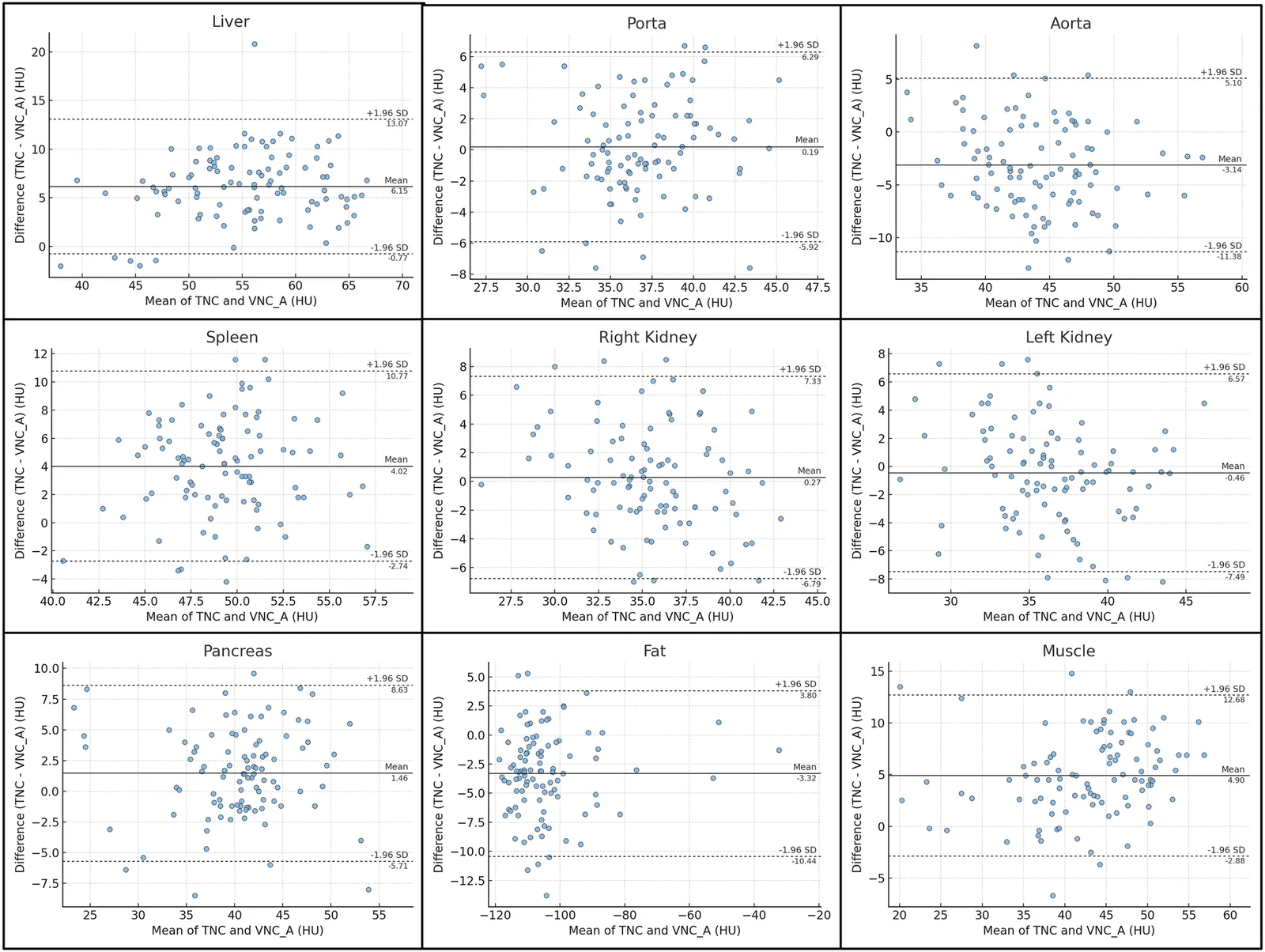
Bland–Altman plots illustrating the agreement between attenuation values (HU) obtained from TNC images and VNC_A for each abdominal organ. The solid black line represents the bias, while the dashed lines indicate the 95% limits of agreement

**Fig. 3:**
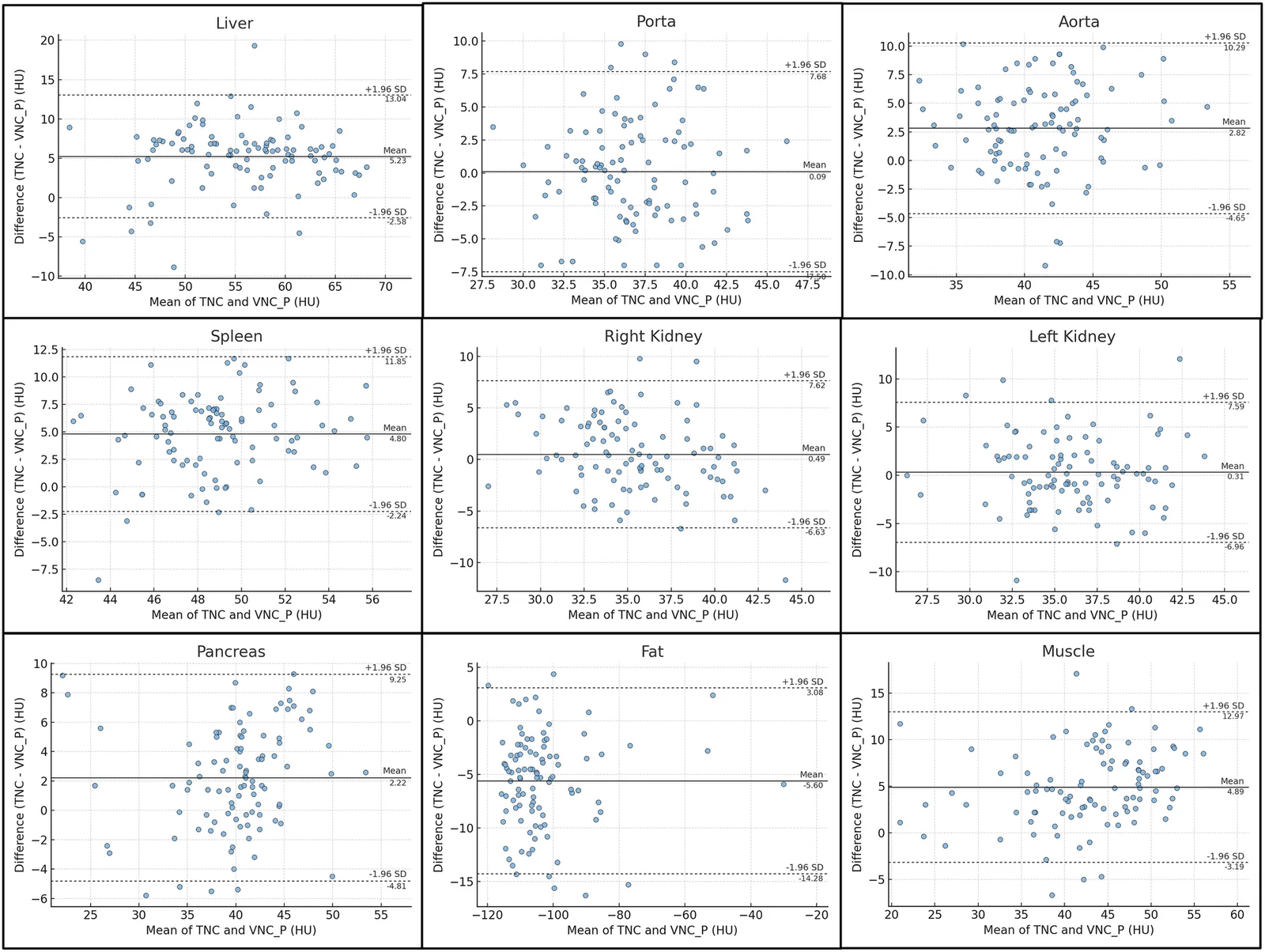
Bland–Altman plots illustrating the agreement between attenuation values (HU) obtained from TNC images and VNC_P for each abdominal organ. The solid black line represents the bias, while the dashed lines indicate the 95% limits of agreement

Attenuation values of TNC, VNC_A, and VNC_P datasets are reported in Table 2; bias and LOA between TNC and both VNC_A and VNC_P are depicted in Figs. 2 and 3, respectively. VNC_A and VNC_P datasets yielded significant differences in attenuation values compared to TNC for several organs. Liver attenuation was significantly higher on TNC (58.4 ± 6.9 HU) compared to both VNC_A and VNC_P (52.2 ± 6.2 HU and 53.1 ± 6.7 HU, respectively; *p* < 0.001), with VNC_P yielding slightly significantly higher values than VNC_A (*p* = 0.005). Similarly, the spleen and pancreas exhibited higher attenuation on TNC compared to both VNC datasets (*p* < 0.001), with a small but significant difference also found between VNC_A and VNC_P for the spleen (*p* = 0.048).

**Table 2:** Attenuation values of TNC, VNC_A, and VNC_P datasets

Hounsfield units						
	TNC	VNC_A	VNC_P	*p*-value (TNC vs VNC_A)	*p*-value (TNC vs VNC_P)	*p*-value (VNC_A vs VNC_P)
Liver	58.4 ± 6.9	52.2 ± 6.2	53.1 ± 6.7	< 0.001	< 0.001	0.005
Porta	36.8 ± 3.8	36.6 ± 3.7	36.7 ± 3.8	1	1	1
Aorta	42.5 ± 4.6	45.7 ± 5.3	39.7 ± 4.8	< 0.001	< 0.001	< 0.001
Spleen	51.3 ± 3.6	47.3 ± 3.3	46.5 ± 3.1	< 0.001	< 0.001	0.048
Right kidney	35.6 ± 3.4	35.3 ± 4.2	35.2 ± 3.4	1	0.540	1
Left kidney	36.1 ± 3.9	36.6 ± 4.7	35.8 ± 4.0	0.630	1	0.058
Pancreas	41.2 ± 6.0	39.8 ± 6.0	39.0 ± 5.5	< 0.001	< 0.001	0.064
Fat	−106.0 ± 13.6	−102.7 ± 13.3	−100.4 ± 13.5	< 0.001	< 0.001	< 0.001
Muscle	45.2 ± 8.3	40.3 ± 7.5	40.3 ± 7.2	< 0.001	< 0.001	1

Data are means ± standard deviations

*TNC* true non-contrast, *VNC_A* virtual non-contrast derived from arterial phase, *VNC_P* virtual non-contrast derived from portal venous phase

The aorta demonstrated significant differences across all three datasets (*p* < 0.001), with VNC_A overestimating (45.7 ± 5.3 HU) and VNC_P underestimating (39.7 ± 4.8 HU) attenuation relative to TNC (42.5 ± 4.6 HU). In contrast, the portal vein and kidneys showed no statistically significant differences in attenuation across any of the reconstructions, except for a borderline non-significant trend in the left kidney between VNC_A and VNC_P (*p* = 0.058).

Muscle attenuation was significantly lower in both VNC datasets compared to TNC (*p* < 0.001), while fat exhibited consistently higher HU values in VNC_A and VNC_P compared to TNC (*p* < 0.001).

### Objective image quality

Comprehensive objective image quality results are reported in Table 3. Signal-to-noise ratio (SNR) was significantly different between the TNC dataset and both VNC_A and VNC_P phases across all evaluated organs. In particular, SNR values were consistently higher in VNC datasets compared to TNC (all *p* < 0.001), with an increase of SNR ranging from 13% in the liver (VNC_A vs TNC: 6.90 ± 1.95 vs 6.09 ± 1.74) to 37% in the aorta (VNC_A vs TNC: 6.01 ± 1.59 vs 4.38 ± 1.02). VNC_A and VNC_P yielded comparable SNR in most organs (*p* ≥ 0.207), except for the aorta, where VNC_A yielded significantly higher SNR (6.01 ± 1.59) than VNC_P (5.35 ± 1.36; *p* < 0.001).

**Table 3:** Objective image quality results of TNC, VNC_A, and VNC_P datasets

SNR						
	TNC	VNC_A	VNC_P	*p*-value (TNC vs VNC_A)	*p*-value (TNC vs VNC_P)	*p*-value (VNC_A vs VNC_P)
Liver	6.09 ± 1.74	6.90 ± 1.95	7.22 ± 2.08	< 0.001	< 0.001	0.207
Porta	3.80 ± 0.94	4.82 ± 1.35	4.97 ± 1.34	< 0.001	< 0.001	0.738
Aorta	4.38 ± 1.02	6.01 ± 1.59	5.35 ± 1.36	< 0.001	< 0.001	< 0.001
Spleen	5.34 ± 1.36	6.25 ± 1.59	6.33 ± 1.61	< 0.001	< 0.001	1
Right kidney	3.69 ± 0.93	4.65 ± 1.21	4.74 ± 1.23	< 0.001	< 0.001	1
Left kidney	3.75 ± 0.94	4.82 ± 1.24	4.85 ± 1.24	< 0.001	< 0.001	1
Pancreas	4.28 ± 1.27	5.25 ± 1.50	5.33 ± 1.59	< 0.001	< 0.001	1

CNR						
	TNC	VNC_A	VNC_P	*p*-value (TNC vs VNC_A)	*p*-value (TNC vs VNC_P)	*p*-value (VNC_A vs VNC_P)
Liver	1.49 ± 1.18	1.62 ± 1.21	1.61 ± 1.17	0.194	0.514	1
Porta	1.05 ± 0.74	0.93 ± 0.71	0.91 ± 0.67	0.191	0.072	1
Aorta	0.77 ± 0.72	1.08 ± 0.89	0.82 ± 0.73	0.003	1	0.012
Spleen	0.88 ± 0.87	1.06 ± 0.96	0.95 ± 0.83	0.005	0.888	0.205
Right kidney	1.17 ± 0.83	1.06 ± 0.85	1.15 ± 0.89	0.351	1	0.558
Left kidney	1.19 ± 0.79	1.04 ± 0.88	1.12 ± 0.84	0.129	0.909	0.651
Pancreas	0.75 ± 0.57	0.77 ± 0.66	0.75 ± 0.68	1	1	1

Data are means ± standard deviations

*CNR* contrast-to-noise ratio, *SNR* signal-to-noise ratio, *TNC* true non-contrast, *VNC_A* virtual non-contrast derived from arterial phase, *VNC_P* virtual non-contrast derived from portal venous phase

Contrast-to-noise ratio (CNR) results showed a different trend: pairwise comparisons for liver, porta, kidneys, and pancreas did not reach statistical significance. VNC_A yielded higher CNR than TNC in the aorta (1.08 ± 0.89 vs 0.77 ± 0.72; *p* = 0.003) and in the spleen (1.06 ± 0.96 vs 0.88 ± 0.87; *p* = 0.005), while VNC_P returned comparable values to TNC (*p* ≥ 0.888). No significant differences were observed between VNC_A and VNC_P for most regions, with the exception of the aorta (1.08 ± 0.89 vs 0.82 ± 0.73; *p* = 0.012).

### Subjective image quality

**Fig. 4:**
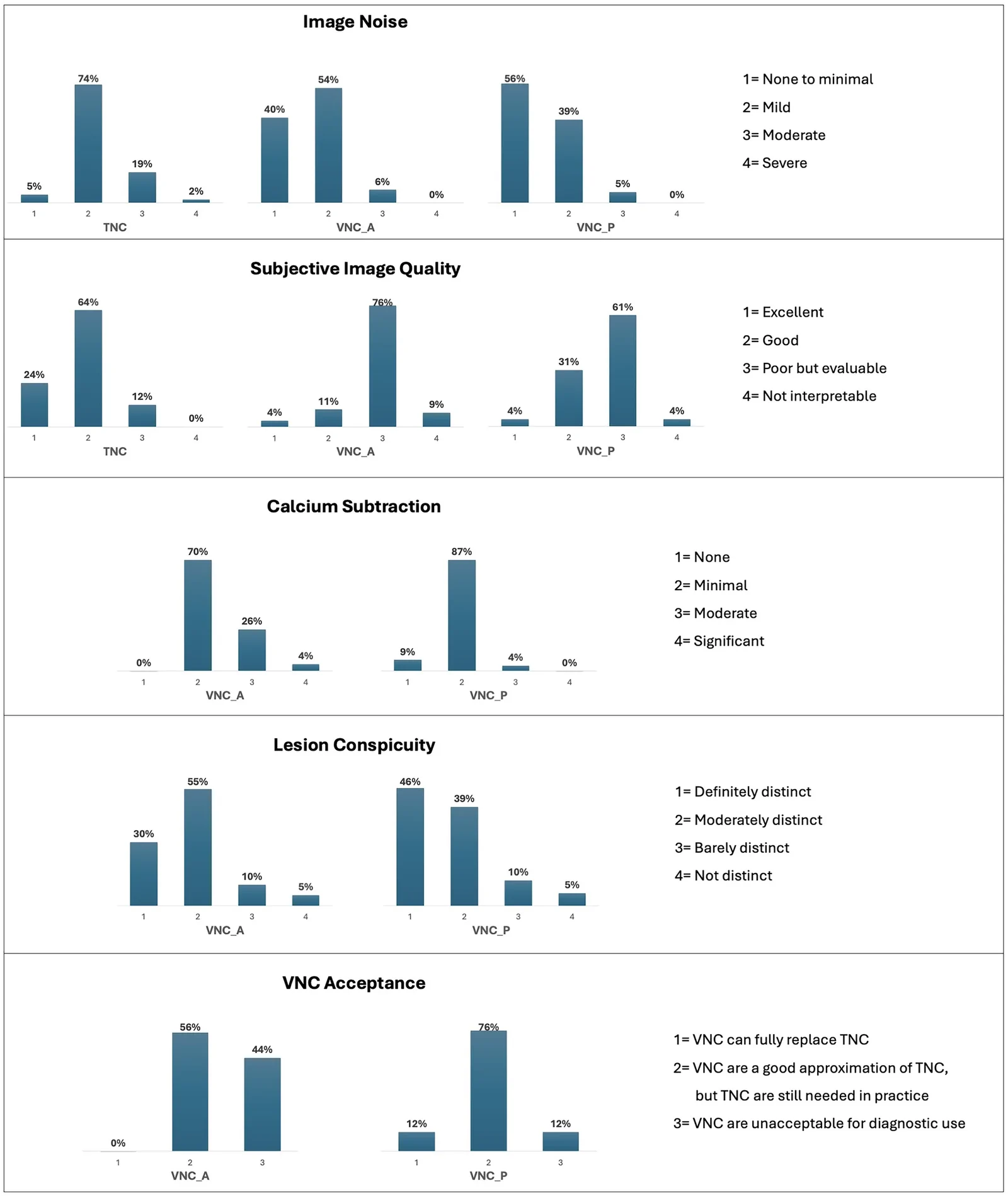
Visual representation of subjective image quality and diagnostic acceptability for TNC, VNC_A, and VNC_P datasets. Bar graphs illustrate the distribution of reader scores across the categories. Likert scale determinants are shown alongside each plot

Comprehensive subjective image quality results are shown in Fig. 4. Overall image quality was rated significantly higher for TNC (median = 2; [IQR, 2–2]) than both VNC_A (median = 3; [IQR, 3–3]) and VNC_P (median = 3; [IQR, 2–3]; all *p* < 0.001). Notably, image quality in TNC was rated excellent in 24% of cases, compared to only 4% for both VNC reconstructions. VNC_P slightly outperformed VNC_A (*p* = 0.024), with a lower proportion of not interpretable cases (4% vs 9%, respectively).

Image noise was comparable between VNC_A (median = 2; [IQR, 1–2]) and VNC_P (median = 1; [IQR, 1–2]; *p* = 0.097) and significantly lower than TNC (median = 2; [IQR, 2–2]; all *p* < 0.001). In particular, noise was graded “none to minimal” in 5% of cases for TNC, versus 40% for VNC_A and 56% for VNC_P. Conversely, TNC was rated as having moderate to severe noise (scores 3–4) in 21% of cases, compared to 6% in VNC_A and 5% in VNC_P.

Calcium subtraction was significantly better in VNC_P (median = 2; [IQR, 2–2]). compared to VNC_A (median = 2; [IQR, 2–3]; *p* < 0.001), while lesion conspicuity did not differ significantly between the two VNC reconstructions (median = 2; [IQR, 1–2]; *p* = 0.141).

Regarding the question of whether VNC reconstructions could replace TNC, most VNC_A and VNC_P cases (56% and 76%, respectively) were graded as a good approximation of TNC, but not a full replacement. Only 12% of VNC_P cases were deemed as a full replacement of TNC. VNC_A was considered unacceptable for diagnostic use in 44% of cases, while VNC_P in 12% of cases (*p* < 0.001).

### Lesions and calcifications analysis

Liver, spleen, and renal lesions demonstrated a substantially higher mean attenuation on TNC (10.5 ± 7.6 HU) compared to both VNC reconstructions derived from arterial (3.2 ± 8.3 HU) and portal venous phases (3.1 ± 7.7 HU), with all comparisons against TNC showing statistical significance (*p* < 0.001), and no difference observed between the VNC phases (*p* = 1). Despite these differences in density, lesion size remained stable across all three image sets (12.2 ± 7.9 mm; *p* = 0.996).

**Fig. 5:**
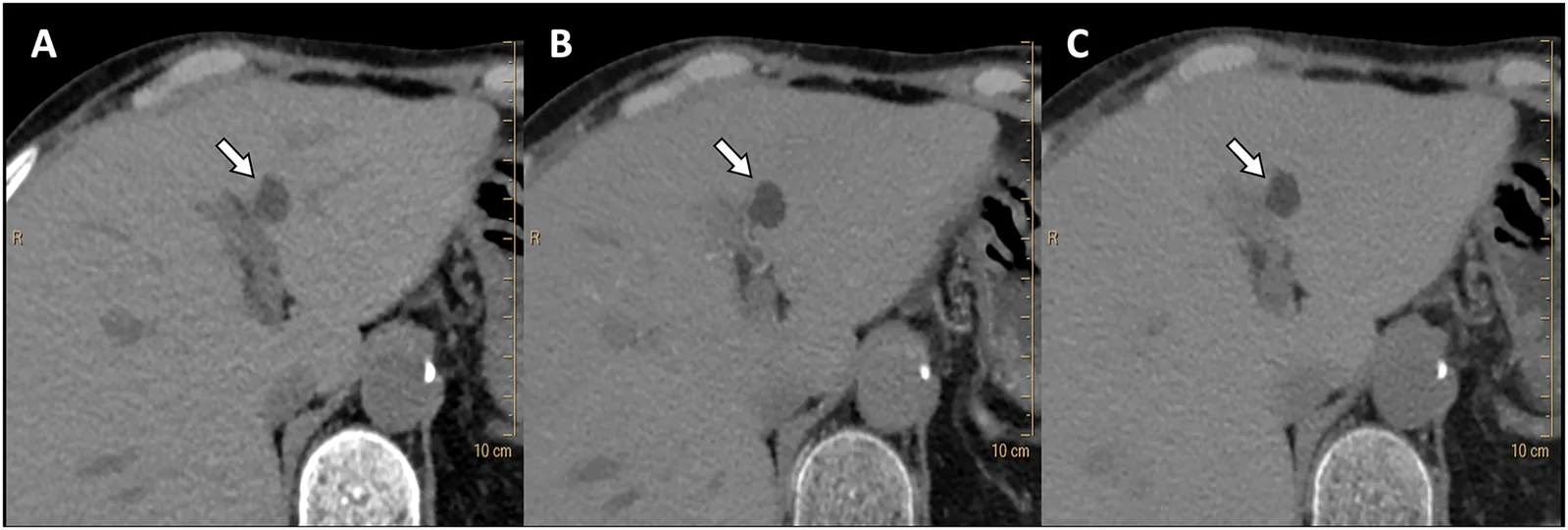
Axial CT images of a 46-year-old female patient with a hypodense liver lesion (arrow): **A** TNC image, **B** VNC_A image, and **C** VNC_P image (W 60 L 360)

**Fig. 6:**
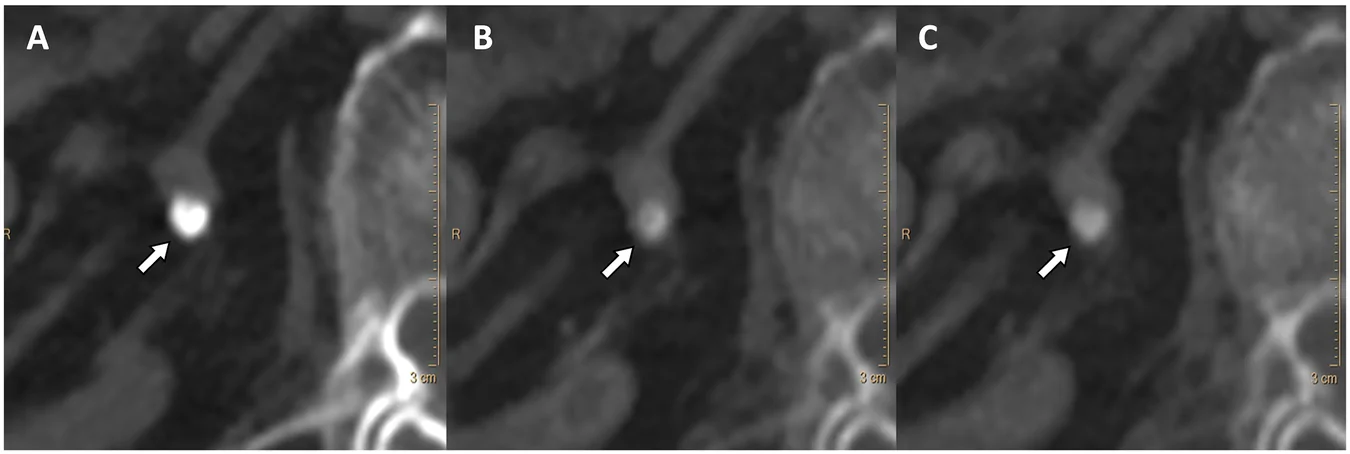
Axial CT images of right renal artery calcification in a 65-year-old male patient (arrow): **A** TNC with average calcification HU values of 885 ± 56, **B** VNC_A image, with average calcification HU values of 284 ± 30, **C** VNC_P image, with average calcification HU values of 310 ± 6. The calcification is less conspicuous in the VNC_A image (W 220 L 900)

For calcifications, mean attenuation was significantly reduced in both VNC_A (354.83 HU) and VNC_P (339.62 HU) compared to TNC (754.83 HU; *p* < 0.001), while the difference between the two VNC phases was not significant (*p* = 0.332). The diameter of calcifications showed a slight but statistically significant reduction in VNC_A (7.3 ± 4.5 mm) compared to TNC (8.0 ± 4.3 mm; *p* < 0.001), whereas no significant difference was found between TNC and VNC_P (8.1 ± 7.5 mm; *p* = 1) or between VNC_A and VNC_P (*p* = 0.513). Examples of the impact of VNC on lesions and calcifications are shown in Figs. 5 and 6, respectively.

### Radiation dose

The mean total DLP was 1583.0 ± 322.9 mGy·cm while the mean DLP of the TNC phase was 532.0 ± 110.4 mGy·cm, resulting in estimated mean ED of 23.7 ± 4.8 mSv and of 7.9 ± 1.6 mSv. In the specific triphasic protocol evaluated in this study, replacement of the TNC phase with VNC resulted in a 33.4% reduction in radiation dose (*p* < 0.001), reflecting the proportional contribution of the TNC phase to the overall protocol dose.

## Discussion

This study evaluated the performance of virtual non-contrast reconstructions derived from arterial (VNC_A) and portal venous (VNC_P) phases compared to true non-contrast (TNC) images. VNC_A demonstrated higher agreement with TNC in terms of attenuation values. Nevertheless, lesion density was significantly reduced in both VNC datasets compared to TNC, although lesion size remained stable across all reconstructions. VNC reconstructions had a substantial impact on calcifications, resulting in density and size reduction. While VNC datasets yielded higher objective image quality, TNC was rated superior in terms of subjective image quality. More importantly, VNC images were mostly considered a good approximation of TNC, but not a full replacement.

VNC reconstructions offer the potential to replace TNC acquisitions, with benefits in terms of reduced radiation exposure and shorter scan time. However, to be clinically viable, especially in abdominal imaging where multiple organs share similar native attenuation values, VNC must ensure reliable attenuation accuracy.

In our investigation, both VNC datasets yielded comparable measurements to TNC only for the portal vein and kidneys. In other tissues, a consistent trend toward attenuation underestimation was observed, although generally within a 10 HU range. These findings are consistent with previous literature documenting a systematic underestimation of attenuation values in VNC images compared to TNC [3, 4, 17]. Notably, attenuation underestimation was also observed for abdominal lesions. This finding is discordant with previous investigations [18], which reported similar attenuation values between TNC and VNC; however, these discrepancies may be attributed to differences in spectral CT technology, and the results may not be directly comparable. Although the magnitude of attenuation underestimation may appear minor, even small differences can impact clinical decision-making, particularly in contexts where HU thresholds guide diagnosis. Conversely, VNC datasets were associated with increased attenuation values in fat. This finding is in line with prior studies, including one using an earlier version of the DLCT scanner, which reported a systematic overestimation of fat density [7]. This trend has also been documented with dual-source CT systems and appears particularly pronounced in adrenal adenomas [19–21], suggesting that it may be an intrinsic limitation of spectral reconstruction techniques rather than a hardware-specific artifact.

The VNC-derived reduction of calcification size and density is in accordance with existing literature on dual-source CT scanners [18, 22]. Similarly, a previous investigation using an earlier DLCT scanner [9] reported significant effects on bone calcium but not on other types of calcifications. The limited ability to accurately subtract calcium encountered across different CT systems is likely due to the similar x-ray attenuation profiles of iodine and calcium, which has a negative impact on spectral differentiation.

The superior objective image quality observed in VNC datasets is offset by a negative impact on image texture, resulting in a blotchier appearance, which is less favored by radiologists: 88% of TNC exams were rated as good or excellent, while most VNC images were considered poor, with a non-negligible portion deemed non-interpretable (9% for VNC_A and 4% for VNC_P). Only 12% of VNC_P images were considered suitable to fully replace TNC, while the majority (56% for VNC_A and 78% for VNC_P) were judged as good approximations but not true substitutes. Our findings are in accordance with several previous investigations, which reported lower subjective image quality for VNC in both parenchymal and vascular settings [22–24]. Notably, the low acceptance of VNC images in our study contrasts with a recent investigation focused on VNC derived from a photon-counting CT scanner (PCCT) [13], where most VNC datasets were deemed fully interchangeable with TNC. This divergence may be partially explained by differences in CT technology. PCCT provides superior spectral separation compared with energy-integrating detector–based systems through energy bin discrimination, which improves material decomposition and iodine subtraction [25]. In contrast, DLCT relies on detector-based separation with partially overlapping spectra, which may contribute to attenuation differences and altered image texture. Additionally, the more focused scope of the PCCT study, limited to renal stones and masses, may have facilitated higher VNC acceptance. In contrast, our broader abdominal assessment may have introduced greater variability and complexity. VNC acceptance might improve when evaluated on more narrowly defined clinical questions, and future research should explore this hypothesis further.

The clinical implications of our findings warrant careful consideration. Despite the substantial radiation dose reduction of a scanning protocol replacing TNC with VNC, further refinements of the VNC algorithm are necessary to substantially improve its agreement with TNC in terms of attenuation values. Alternatively, adjusted diagnostic thresholds may be established and adopted when interpreting VNC images instead of TNC, particularly in scenarios where absolute HU values guide diagnosis. The beneficial impact of replacing TNC with VNC would be particularly effective in the oncologic setting, where patients often undergo multiple CT examinations: scanning protocols may include TNC images at baseline while relying on VNC reconstructions for follow-up studies. It also remains to be determined whether the small attenuation differences observed with VNC have a clinical impact, especially considering that HU variability is already known to occur across different CT vendors, scanner generations, and reconstruction kernels [26]. Within this context, minor VNC-related discrepancies may fall within the range of routine inter-scanner variability.

This study has some limitations that need to be addressed. First, this was a single-center study focused on a vendor-specific technology; therefore, our results may not be directly transferable to other institutions and to different spectral CT techniques, such as fast kV-switching and dual-source CT systems. Direct comparisons of the three different spectral approaches are advisable to better understand their reliability in generating VNC images of the abdomen. Second, although readers were blinded to the reconstruction type, we cannot fully exclude that the different image texture of VNC images may have introduced a scoring bias. Third, this investigation excluded patients with severe steatosis, cirrhosis, and diffuse lesions, aiming at removing potentially confounding factors in ROIs placement. Nevertheless, the VNC datasets require validation also in patients with diffuse liver disease to be fully implemented in routine clinical practice.

In conclusion, VNC datasets significantly affect attenuation values in abdominal imaging, with consistent underestimation across most structures and a significant impact on calcium density and size; additionally, despite improving SNR and CNR, it remains inferior in subjective image quality compared to TNC. Therefore, VNC should not be used as a prospective replacement for TNC in routine protocols, while it may serve as a valuable retrospective problem-solving tool when TNC is unavailable, provided it is interpreted with caution. Thus, VNC represents a valuable approximation but cannot fully replace TNC yet, and further refinements are needed to achieve large-scale clinical adoption.

## Supplementary information

Supplementary information

## Abbreviations

CNR: Contrast-to-noise ratio
DLCT: Dual-layer spectral detector CT
DLP: Dose-length product
ED: Effective dose
ICC: Intraclass correlation coefficient
LOA: Limit of agreement
PCCT: Photon-counting CT
SNR: Signal-to-noise ratio
TNC: True non-contrast images
VNC: Virtual non-contrast images
VNC_A: Virtual non-contrast images derived from arterial phase
VNC_P: Virtual non-contrast images derived from portal venous phase
