# Supplementary material for: Virtual non-contrast images from dual-layer spectral CT: comparison with true non-contrast across abdominal structures
Source: Eur Radiol. 2026 Jun 3;36(10):7718–30. doi: 10.1007/s00330-026-12642-0 (PMC13574884; doi:10.1007/s00330-026-12642-0)
Supplement: Supplementary file 1 [file 330_2026_12642_MOESM1_ESM.pdf]

ELECTRONIC SUPPLEMENTARY MATERIAL

Virtual Non-Contrast Images from Dual-Layer Spectral CT: Comparison with True Non-Contrast Across Abdominal Structures

Eur Radiol (2026) De Santis D, Del Gaudio A, Masci B et al.

Supplemental Table 1

4-point Likert scale determinants for subjective image quality assessment.

Score	Overall Image Quality	Image Noise	Calcium Subtraction	Lesion Conspicuity
1	Excellent	None to minimal	None	Not distinct
2	Good	Mild	Minimal	Barely distinct
3	Poor but evaluable	Moderate	Moderate	Moderately distinct
4	Not interpretable	Severe	Significant	Definitely distict
